# Brain metabolic pattern analysis using a magnetic resonance spectra classification software in experimental stroke

**DOI:** 10.1186/s12868-016-0328-x

**Published:** 2017-01-13

**Authors:** Elena Jiménez-Xarrié, Myriam Davila, Ana Paula Candiota, Raquel Delgado-Mederos, Sandra Ortega-Martorell, Margarida Julià-Sapé, Carles Arús, Joan Martí-Fàbregas

**Affiliations:** 1 Stroke Unit, Department of Neurology, Hospital de la Santa Creu i Sant Pau, IIB-Sant Pau, Sant Antoni Maria Claret 167, 08025 Barcelona, Spain; 2 Departament de Bioquímica i Biologia Molecular, Unitat de Biociències, Edifici C, Universitat Autònoma de Barcelona, 08193 Cerdanyola del Vallès, Spain; 3 Centro de Investigación Biomédica en Red en Bioingeniería, Biomateriales y Nanomedicina (CIBER-BBN), 08193 Cerdanyola del Vallès, Spain; 4 Institut de Biotecnologia i de Biomedicina, Universitat Autònoma de Barcelona, 08193 Cerdanyola del Vallès, Spain; 5 Department of Applied Mathematics, Liverpool John Moores University, Byrom Street, Liverpool, L3 3AF UK

**Keywords:** Magnetic resonance spectroscopy, Metabolomics, Pattern recognition, Stroke, Animal model

## Abstract

**Background:**

Magnetic resonance spectroscopy (MRS) provides non-invasive information about the metabolic pattern of the brain parenchyma in vivo. The SpectraClassifier software performs MRS pattern-recognition by determining the spectral features (metabolites) which can be used objectively to classify spectra. Our aim was to develop an Infarct Evolution Classifier and a Brain Regions Classifier in a rat model of focal ischemic stroke using SpectraClassifier.

**Results:**

A total of 164 single-voxel proton spectra obtained with a 7 Tesla magnet at an echo time of 12 ms from non-infarcted parenchyma, subventricular zones and infarcted parenchyma were analyzed with SpectraClassifier (http://gabrmn.uab.es/?q=sc). The spectra corresponded to Sprague-Dawley rats (healthy rats, n = 7) and stroke rats at day 1 post-stroke (acute phase, n = 6 rats) and at days 7 ± 1 post-stroke (subacute phase, n = 14). In the Infarct Evolution Classifier, spectral features contributed by lactate + mobile lipids (1.33 ppm), total creatine (3.05 ppm) and mobile lipids (0.85 ppm) distinguished among non-infarcted parenchyma (100% sensitivity and 100% specificity), acute phase of infarct (100% sensitivity and 95% specificity) and subacute phase of infarct (78% sensitivity and 100% specificity). In the Brain Regions Classifier, spectral features contributed by myoinositol (3.62 ppm) and total creatine (3.04/3.05 ppm) distinguished among infarcted parenchyma (100% sensitivity and 98% specificity), non-infarcted parenchyma (84% sensitivity and 84% specificity) and subventricular zones (76% sensitivity and 93% specificity).

**Conclusion:**

SpectraClassifier identified candidate biomarkers for infarct evolution (mobile lipids accumulation) and different brain regions (myoinositol content).

**Electronic supplementary material:**

The online version of this article (doi:10.1186/s12868-016-0328-x) contains supplementary material, which is available to authorized users.

## Background

Magnetic resonance spectroscopy (MRS) is a technique that allows obtaining metabolic information of the brain parenchyma in vivo non-invasively, either in preclinical or clinical studies [1]. However, MRS is not used routinely in the diagnosis and management of stroke patients probably because metabolic biomarkers that can help to make clinical decisions have been scarcely reported [2].

Metabolites have defined chemical shifts [parts per million (ppm)] in the magnetic resonance (MR) spectrum, so each metabolite can be individually identified and quantified. The quantification can be performed based on several approaches: peak heights or ratios, integrals based on resonance fittings, or quantification using software programs like LCmodel [3], jMRUI accessible routines [4] or TARQUIN [5].

A multivariate statistical analysis of the whole MR spectrum is more appropriate than individual feature analysis as it gives information about many metabolites simultaneously. The increase/decrease of some of these metabolites may be characteristic of a certain condition and at the same time distinguish this condition from others. This allows performing a classifier based on features (peak heights of typical resonances) which can be useful to assign unknown spectra to a condition.

A classifier based on multivariate statistical analysis may be an unbiased way of categorizing spectral data as it is a method with a minimum requirement for prior hypothesis to be made. This may be especially relevant when there is no prior knowledge of which are the metabolites to be considered relevant, for example in a certain pathology or as a consequence of a treatment.

SpectraClassifier (http://gabrmn.uab.es/?q=sc) [6] is a pattern-recognition software that applies multivariate statistical analysis for classifying MR spectra. It performs a supervised analysis, where the researcher knows a priori the real class to which each MR spectrum (from now on called case) belongs to. Thus, the researcher predefines the classes and assigns each case (by adding a *label*) to its corresponding class.

The software uses a set of *labelled* cases (the training set) to produce a predictive mathematical model, which is the classifier, by identifying the datapoint intensities that differ most among the classes (features). The classifier is then validated by assessing its predictive accuracy not only for the training set cases, but also when similar new cases (*labelled* or not) are analyzed (an independent test set) [7]. So far, SpectraClassifier has been applied to evaluate brain cancer either with preclinical [8] or clinical [9, 10] MRS data.

Multivariate statistical analysis is increasingly being applied to MRS data in different diseases [11]. In stroke, multivariate statistical analysis has been applied to the MRS metabolic analysis of plasma and urine samples from stroke patients [12] and metabolite extracts of infarcted tissue in a rat model of ischemic stroke [13]. However, there is no multivariate analysis of in vivo MRS data from specific regions of interest in the stroke-afflicted brain. Our aim was to find, in a rat model of focal ischemic stroke, surrogate biomarkers of the infarct evolution and for different brain regions including the subventricular zones (SVZ) of the lateral ventricles, which are a known neurogenic niche in the adult mammalian brain [14] affected by stroke [15, 16].

In our study, we provide proof-of-concept of two different classifiers generated using SpectraClassifier: (a) Infarct Evolution Classifier (to differentiate among non-infarcted parenchyma, acute phase of infarct and subacute phase of infarct and (b**)** Brain Regions Classifier (to differentiate among non-infarcted parenchyma, SVZ and infarcted parenchyma).

## Methods

### Animals

A total of twenty-seven male Sprague-Dawley rats (Charles River Laboratories, L’Arbresle, France) of approximately 10 weeks of age and 306 ± 39 g of weight were used. MR spectra from 24 rats were obtained retrospectively from a previous study performed on a rat model of focal ischemic stroke [17] while three additional rats with available spectroscopic information were added, not included in the previous study due to incomplete data but belonging to the same protocol.

Rats were divided into the following groups: healthy rats (n = 7), day 1 post-stroke rats (acute phase of infarct, n = 6) and day 7 ± 1 post-stroke rats (subacute phase of infarct, n = 14), according to the temporal classification of Pitkonen et al. [18]. Rats from the last group were analyzed at two time points (acute and subacute phase of infarct).

The rat model of focal ischemic stroke was the 90 min right middle cerebral artery occlusion (MCAO) with the intraluminal filament [19] which results in a large infarct (250 ± 82 mm^3^ measured at day 1 post-stroke in T_2_-weighted images). After surgery, analgesia (Metacam, Boheringer Inghelm GmbH, Inghelm, Germany, 1.0 mg/kg body weight) was administered subcutaneously. Rats were housed at 25 ± 1 °C and were kept on a 12/12 h light/darkness cycle with ad libitum access to food and water.

### Magnetic resonance studies

In vivo magnetic resonance studies were carried out at the joint nuclear magnetic resonance facility of the *Universitat Autònoma de Barcelona* and *Centro de Investigación Biomédica en Red* – *Bioingeniería, Biomateriales y Nanomedicina* (CIBER-BBN) (Cerdanyola del Vallès, Spain), Unit 25 of NANBIOSIS, in a 7 Tesla horizontal magnet (BioSpec 70/30, Bruker BioSpin, Ettlingen Germany) equipped with actively shielded gradients (B-GA12 gradient coil inserted into a B-GA20S gradient system). For signal reception, a rat brain phased array coil was used, actively decoupled from a 72 mm inner diameter volume resonator. During exploration the rats were anesthetized with 1–2% isoflurane (IsoVet, Braun VetCare, Spain). Breathing and temperature were constantly monitored (SA Instruments, Inc, New York, USA).

**Fig. 1:**
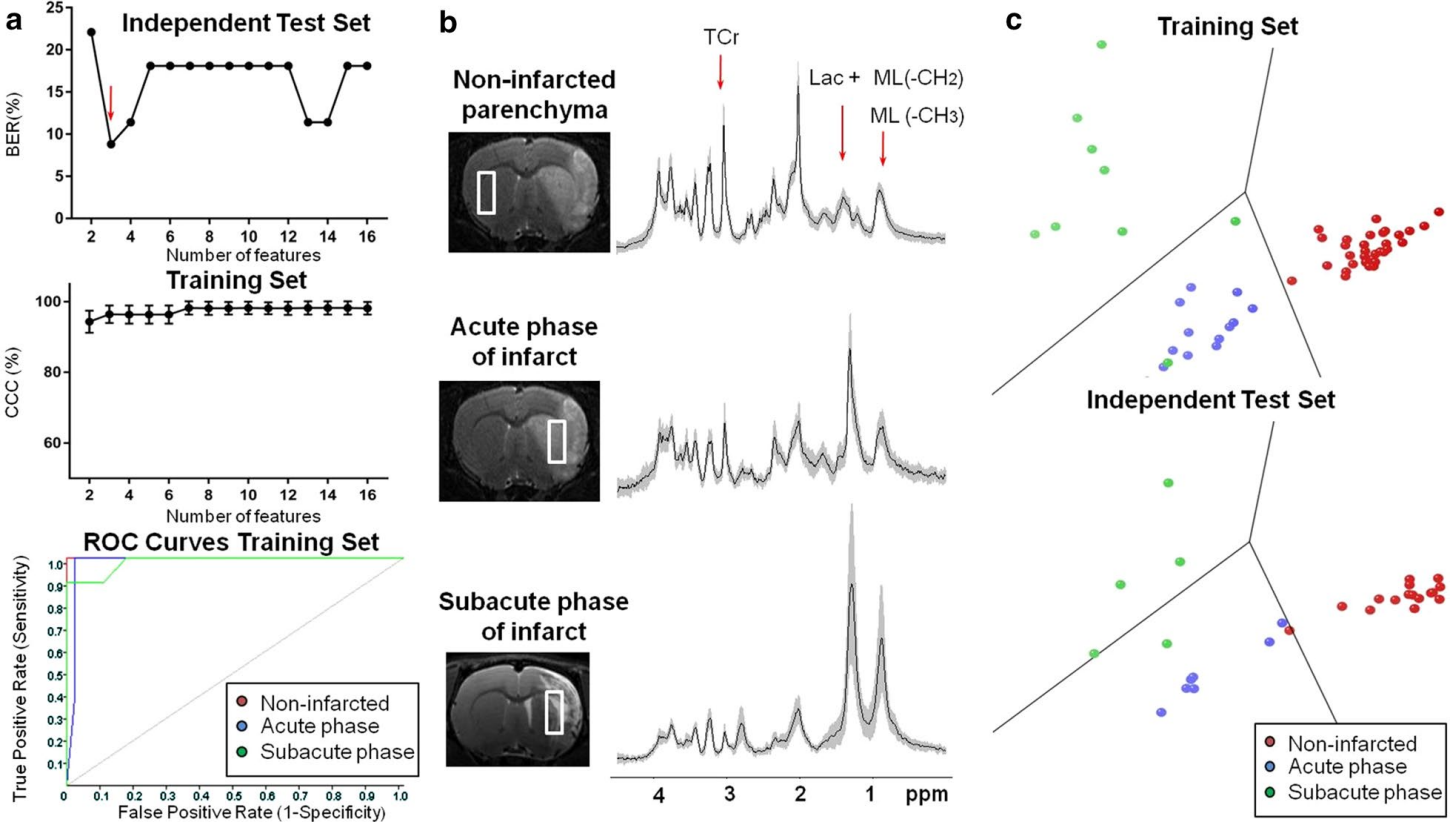
Results from the Infarct Evolution Classifier. **a** Analysis of the Balanced Error Rate (BER) of the independent test set, the correctly classified cases (CCC) and the plot of the three ROC curves of the training set. The best performance was achieved using three features (*red arrow*). **b** Image of the voxel position and the mean spectrum ± SD (in *gray shading*) of the training set for each class with the approximate position of the features selected by the SFFS method indicated by *red arrows* (see also Table 1). **c** 2D Fisher’s LDA latent space representing the classification in the training set and the independent test set using three features

**Fig. 2:**
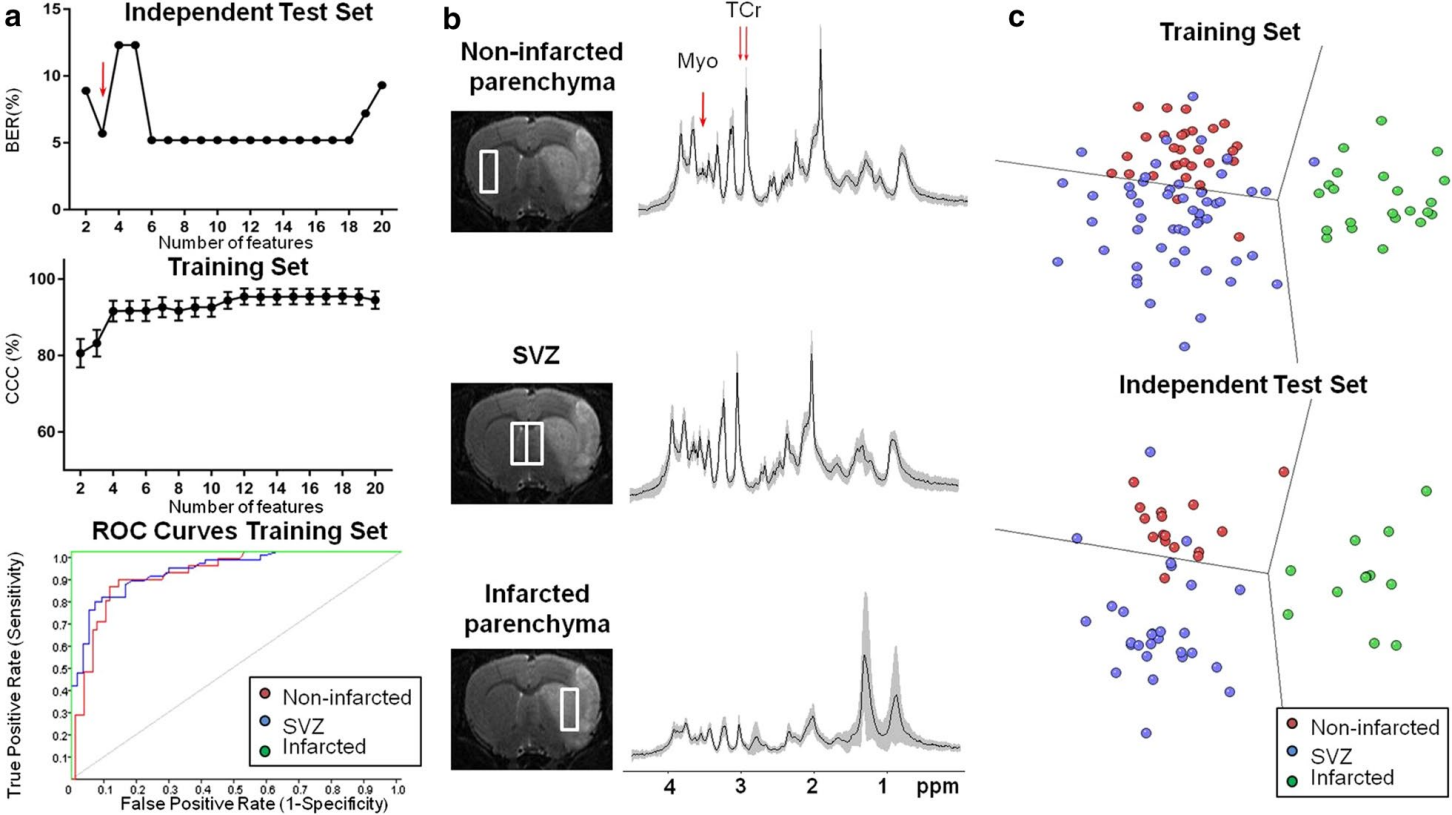
Results from the Brain Regions Classifier. **a** Analysis of the Balanced Error Rate (BER) of the independent test set and the correctly classified cases (CCC) and the plot of the three ROC curves of the training set. The best performance was achieved using three features (*red arrow*). **b** Image of the voxel position and the mean spectrum ± SD (in *gray shading*) of the training group for each class with the position of the features selected by the SFFS method indicated approximately by red arrows (see also Table 3). **c** 2D Fisher’s LDA latent space representing the classification in the training set and the independent test set using three features

T_2_-weighted images were used for infarct volume measurement [17] and MRS voxel positioning (a schematic representation is shown in Figs. 1b, 2b). These were acquired using a rapid acquisition with a relaxation-enhancement sequence. The acquisition parameters were: orientation = coronal plane, echo train length = 8, field of view = 32 × 32 mm, matrix size = 256 × 256 (125 × 125 μm/pixel), number of slices = 30, slice thickness = 0.5 mm, interslice distance = 0.1 mm, repetition time/effective echo time = 4560/60 ms, number of acquisitions = 4, total acquisition time = 7 min and 17 s.

MR spectra were acquired from rectangular voxels of 18 µl (2 × 2 × 4.5 mm) using point-resolved spectroscopy localization and variable pulse power and optimized relaxation delay water suppression. The acquisition parameters were: repetition time/ echo time = 1800/12 ms, spectral width = 13.34 ppm (4006.41 Hz), number of acquisitions = 256; total acquisition time = 7 min and 48 s.

A total of 164 MR spectra were obtained from 27 Sprague Dawley rats. The spectra corresponded to:

- 34 Infarcted parenchyma spectra: 6 spectra were obtained from day 1 post-stroke rats and 28 spectra from days 7 ± 1 post-stroke rats analyzed at two different time points (the acute and the subacute phase of infarct).
- 82 SVZ spectra from both cerebral hemispheres: 14 spectra were obtained from healthy rats, 12 spectra from day 1 post-stroke rats and 56 spectra from day 7 ± 1 post-stroke rats analyzed at two different time points (the acute and the subacute phase of infarct. Due to the dimensions of the acquisition voxel, its 2 mm thickness will unavoidably produce contributions from outside the SVZ region (i.e. nearby striatum, cerebrospinal fluid from the ventricle). In this respect, SVZ should be taken to mean “SVZ enriched” brain parenchyma MRS pattern.
- 48 Non-infarcted parenchyma spectra: 14 spectra were obtained from both cerebral hemispheres in healthy rats, 6 spectra from non-infarcted parenchyma from day 1 post-stroke rats and 28 spectra from non-infarcted parenchyma from day 7 ± 1 post-stroke rats analyzed at two different time points (the acute and the subacute phase of infarct).

### MR spectra processing

MR spectra were processed using Topspin 1.3 software (Bruker Daltonik Gmbh, Rheinstetten, Germany). Fourier transformation was applied with previous 4 Hz line broadening. Manual zero- and first-order phase corrections were applied and the chemical shifts were referenced to total creatine (TCr) peak maxima at 3.03 ppm. MRS data peak heights were normalized to unit length to the contralateral area (UL_2_CA) (non-infarcted parenchyma) according to Eq. 1 for the region between 0 and 4.5 ppm. This region was digitized with 1383 complex datapoints and exported as ASCII files.

1 $$UL2_ {CA} = \;\frac{{h_{real} }}{{\sqrt {\sum\nolimits_{0}^{4.5} {(h_{real} CA)^{2} } } }}\cdot100$$

Equation 1: Unit length normalization to the contralateral area (UL_2_CA). h_real_ refers to the peak heights at each datapoint in the real part of the acquired spectra and h_real_CA refers to the peak heights between 0 and 4.5 ppm from the contralateral area spectra of each animal.

### Supervised pattern-recognition analysis

The Pattern-Recognition analysis was performed with SpectraClassifier v3.1.1 software (http://gabrmn.uab.es/?q=sc). We used the available cases to design two classifiers, each including three classes.

1. The Infarct Evolution Classifier: comparison among 48 non-infarcted parenchyma spectra (32 training set and 16 test set), 20 acute phase of infarct spectra (13 training set and 7 test set) and 14 subacute phase of infarct spectra (9 training set and 5 test set). The processed data vectors used to perform the Infarct Evolution Classifier are provided as additional files: training set (Additional file 1) and test set (Additional file 2).
2. The Brain Regions Classifier: comparison among 48 non-infarcted parenchyma spectra (32 training set and 16 test set), 82 SVZ spectra (54 training set and 28 test set) and 34 infarcted parenchyma spectra (22 training set and 12 test set). The processed data vectors used to perform the Brain Regions Classifier are provided as additional files: training set (Additional file 3) and test set (Additional file 4).

Data splitting into the training or the test set was performed by an investigator (MD) different from the one acquiring spectra (EJX) and blinded to the quality of those spectra. Approximately 2/3 of the spectra were assigned by MD to the training set and 1/3 of the spectra to the independent test set [20]. This data splitting process was based on the spectra organized by a unique animal identification number (ID, lower numbers indicating animals incorporated to the protocol earlier). Once the total number of spectra to be allocated in each training dataset was decided, spectra of the lower ID number rats contributed to training set and the remaining high ID number contributed all to the test set. This “chronological” methodology was used as it would emulate a real machine learning protocol were new prospective cases could be added continuously to the test set. The final allocation of each case in the training or the test set can be seen in the additional file identified in the xml file.

Feature selection was performed by sequential forward feature selection (SFFS), based on the criterion of correlation-based feature subset selection. The classifier was created using Fisher’s linear discriminant analysis (LDA), and the number of selectable features was set from 2 to 20. Ideally, the final number of selected features should be approximately 1/3 of the number of cases in the smallest size class of the training set [21]. It took approximately 15 min for each classifier to perform feature selection using SpectraClassifier v3.1.1 on a core i5-6600 K@3.5 GHz with 16.38 GB of RAM. The selection of the overall best descriptive performance of the classifier was determined by the number of features that provided the highest correctly classified cases (CCC)-value in the training set by Bootstrapping. Bootstrapping was performed by 1000 times random sampling with replacement from the original dataset.

The final number of features used for classification was selected based on the criterion of the lowest balanced error rate (BER)-value (Eq. 2) in the independent test set. The BER-value is the average of the proportion of misclassified cases in each class weighted by the size of the class [8] which is an overall measure of the predictive accuracy.

2 $$BER = \;\frac{1}{3}\cdot\left[ {\left( {\frac{misclassified\;cases}{total\;cases}} \right)_{CL1} + \left( {\frac{misclassified\;cases}{total\;cases}} \right)_{CL2} + \left( {\frac{misclassified\;cases}{total\;cases}} \right)_{CL3} } \right]\cdot100$$

Equation 2: Balanced Error Rate (BER) for a three class classifier where CL1, CL2 and CL3 correspond to the first, second and third class respectively.

### Statistics

The multivariate statistical methods integrated in SpectraClassifier were used to calculate the classifier based on Fisher’s LDA [6].

For further analysis of the predictive accuracy for each class, data of the training set were dichotomized to one class compared to the other two classes combined. The analyses performed were: (1) sensitivity, specificity, positive predictive value (PPV) and negative predictive value (NPV) calculated with Vassar stats (www.vassarstats.net) using the confusion matrix values provided by SpectraClassifier and (2) the area under the curve (AUC) of the receiver operating characteristic (ROC) provided by SpectraClassifier.

The normality of the data distribution of the individual selected features was assessed with Kolmogorov–Smirnov test and variance homogeneity with Levene’s test. The results obtained with Kolmogorov–Smirnov test showed that not all features followed a normal distribution. Accordingly, the non-parametric Kruskall–Wallis test for independent samples was used to evaluate differences among classes. Post-hoc pairwise comparisons were performed using SPSS 22.0 software (SPSS Inc., Chicago, IL, USA). The α-level for statistical significance was 0.05 with a confidence interval of 95%. Home-written R software scripts were used for the mean spectrum ± standard deviation (SD) representation.

## Results

### Infarct Evolution Classifier

The Infarct Evolution Classifier was performed using from 2 to 16 features. The training set showed an increasing CCC-value as more features were used for the classification. However, the first minimum BER-value (8.8%) in the independent test set was achieved with three features. This number of features corresponded to a CCC-value of 96.4 ± 2.5% in the training set (Fig. 1a).

The three selected features with their tentative resonance assignment according to the literature [22] are shown in Table 1 and Fig. 1b. The selected features (in their selection order number) correspond to individual spectral datapoints: the first containing overlapping lactate and the methylene of the fatty acyl chain of mobile lipids [Lac + ML(–CH_2_)] at 1.33 ppm, the second to the N-methyl of TCr at 3.05 ppm and the third to the methyl of the fatty acyl chain of mobile lipids [ML(–CH_3_)] at 0.85 ppm [23].

**Table 1:** Features selected for the Infarct Evolution Classifier, and the metabolites tentatively assigned to those features

Feature selection order number	Feature chemical shift (ppm)	Assigned metabolite	Non-infarcted parenchyma*	Acute phase post-stroke*	Subacute phase post-stroke*	p value^†^
1	1.33	Lac + ML(–CH_2_)	2.78 (2.38–3.21)	7.39 (6.67–8.90)	19.88 (10.38–22.11)	p < 0.01
2	3.05	TCr	3.83 (3.42–4.18)	1.96 (1.63–2.36)	2.01 (1.62–2.31)	p < 0.01
3	0.85	ML(–CH_3_)	3.32 (3.09–3.95)	3.02 (2.33–4.27)	12.30 (8.39–15.52)	p < 0.01

* Median (interquartile range) of UL_2_CA normalized peak height values for each feature in the training set

^†^p value resulting from the Kruskall–Wallis test of the comparison of the selected feature among the three classes of the training set

The classifier was trained with three features (Fig. 1c). The results for each class are shown on Table 2. The ability of this classifier to determine whether a stroke corresponds to the acute or the subacute phase of infarct showed a higher sensitivity in the acute phase class (100%) compared to the subacute phase class (78%). However, in the latter, the specificity was 100% which gives an optimum PPV (100%).

**Table 2:** Infarct Evolution Classifier predictive accuracy analysis in the training set

Training set	Sensitivity	Specificity	PPV	NPV	AUC value
Non-infarcted parenchyma	100% (32/32)	100% (22/22)	100% (32/32)	100% (22/22)	1.00 ± 0.00
Acute phase of infarct	100% (13/13)	95% (39/41)	87% (13/15)	100% (39/39)	0.98 ± 0.00
Subacute phase of infarct	78% (7/9)	100% (45/45)	100% (7/7)	96% (45/47)	0.98 ± 0.04

Sensitivity, specificity, positive predictive value (PPV), negative predictive value (NPV) and the mean ± standard error area under curve (AUC) value of the receiver operating curve (ROC) of the dichotomization of each class compared to the other two classes combined. Results are given in percentage with the number of spectra within parentheses. Total number of spectra in the training set (n = 54) corresponded to non-infarcted parenchyma (n = 32), acute phase of infarct (n = 13) and subacute phase of infarct (n = 9).

Median and interquartile ranges of the peak height values of the selected spectral features for each class are shown on Table 1. The post-hoc analysis of both TCr and Lac + ML(–CH_2_) features showed statistical difference (p < 0.01) when comparing non-infarcted parenchyma class with the other two classes. In Lac + ML(–CH_2_), there was no significant difference between the acute and the subacute phase of infarct classes, probably due to higher variability in the subacute phase of infarct. Accordingly, ML(–CH_3_) was the only metabolite resonance that increased significantly (p < 0.01) between the acute and the subacute phase of infarct.

### Brain Regions Classifier

For the Brain Regions Classifier, spectra from brain parenchyma at different time-points post-stroke (non-infarcted parenchyma, acute and subacute phase of infarct) were combined into the same class.

The Brain Regions Classifier was performed using from 2 to 20 features. In this classifier, the first minimum BER-value of the independent test set (5.7%) was achieved using three features for classification (Fig. 2a). This number of features corresponded to a CCC-value of 83.2 ± 3.5% in the training set. Another minimum BER-value was achieved with 6 features (5.5%) which corresponded to a better CCC-value of 91.7 ± 2.7%. However, the classifier was trained using three features as the results shown by the BER-value were similar. Moreover, when the spectral features selected in the fourth and fifth position were added, the classification was worse, as the BER-value rose to 12.3%. Accordingly, adding spectral features might be a source of overtraining for the final classifier [24].

The selected spectral features corresponding to the first and the third selection order number were very close datapoints in the MR spectrum (3.04 and 3.05 ppm) and were related to the same resonance (TCr) as in the Infarct Evolution Classifier. Still, it should be mentioned that TCr is contributed by resonances from several metabolites, with slightly different chemical shifts: phosphocreatine, creatine, γ-aminobutyric acid (GABA), glutathione, lysine in small molecular weight proteins. Accordingly, changes in their relative contributions could make two close datapoint heights relevant for classification performance. The second selected feature (3.62 ppm) was related to myoinositol (Myo) (Table 3; Fig. 2b).

**Table 3:** Features selected for the Brain Regions Classifier and the metabolites tentatively assigned to those features

Feature selection order number	Feature chemical shift (ppm)	Assigned metabolite	Non-infarcted parenchyma*	SVZ*	Infarcted parenchyma*	p value†
1	3.05	TCr	5.80 (5.31–6.04)	5.74 (5.26–6.44)	2.43 (2.01–3.32)	p < 0.01
2	3.62	Myo	2.73 (2.31–2.93)	3.45 (3.15–3.91)	1.61 (1.41–2.01)	p < 0.01
3	3.04	TCr	6.92 (6.47–7.37)	6.91 (6.38–7.70)	2.84 (1.95–3.80)	p < 0.01

* Median (interquartile range) for the UL_2_CA normalized peak height values for each selected spectral feature using the SFFS method in the training set

^†^p value resulting from the non-parametric Kruskall–Wallis test of the comparison of the selected spectral feature among the three classes of the training set

Results for each class are shown on Table 4. The classifier showed good predictive values for the infarcted parenchyma class in the training set (100% of sensitivity and 98% of specificity). In this classifier the misclassified cases were found to belong to the non-infarcted parenchyma and the SVZ, which show similar MR spectral patterns (Fig. 2b). Thus, when the ability of the classifier to identify the SVZ was analyzed, it had moderate sensitivity (76%) but good values of specificity (93%) and a good PPV (91%).

**Table 4:** Brain Regions Classifier predictive accuracy analysis in the training set

Training set	Sensitivity	Specificity	PPV	NPV	AUC
Non-infarcted parenchyma	84% (27/32)	84% (64/76)	69% (27/39)	93% (64/69)	0.90 ± 0.06
SVZ	76% (41/54)	93% (50/54)	91% (41/45)	79% (50/63)	0.92 ± 0.04
Infarcted parenchyma	100% (22/22)	98% (84/86)	92% (22/24)	100% (84/84)	1.00 ± 0.00

Sensitivity, specificity, positive predictive value (PPV), negative predictive value (NPV) and the mean ± standard error area under curve (AUC) value of the receiver operating curve (ROC) of the dichotomization of each class compared to the other two classes combined. Results are given in percentage with the number of spectra within parentheses. Total number of spectra in the training set (n = 108) corresponded to non-infarcted parenchyma (n = 32), SVZ (n = 54) and infarcted parenchyma (n = 22).

When the features selected by the classifier were analyzed individually, a decreased TCr (p < 0.01) allowed the differentiation of the infarcted parenchyma class compared to the other two classes. For Myo, there was a statistically significant difference among the three classes (p < 0.01). It was increased in the SVZ and decreased in the infarcted parenchyma with respect to the non-infarcted parenchyma.

## Discussion

The two classifiers developed with SpectraClassifier have good predictive values and a robust independent test set performance, which suggests that they have discriminatory power. The selected metabolites used for class discrimination may be of interest as potential biomarkers to monitor infarct evolution or to evaluate whether a specific MRS-based biomarker for the SVZ exists.

Numerous studies have utilized MRS to analyze metabolic changes in experimental stroke. Most literature focuses on metabolites of interest for the pathology, such as lactate, which is related to anaerobic metabolism onset and *N*-acetyl aspartate (NAA), which is related to neuron viability [25].

In the acute phase of stroke, several dynamic metabolic changes occur. Studies in a permanent MCAO rat model showed that increases in lactate and decreases in NAA as well as increases in glutamate and taurine are already detectable at 1 h post-stroke [26]. Recent studies performed approach to a complete analysis of the whole metabolome. In brain tissue extracts from a permanent MCAO rat compared with the sham group at day 1 post-stroke, an increase in glucose and decreases in glutamate, glutamine, aspartate, myoinositol, GABA, taurine, malate, fumarate, acetate, phosphocreatine, and purine and pyrimidine metabolites such as inosine, hypoxanthine, xanthine and uracil were detected. However, in this work neither NAA nor lactate were selected as metabolites that changed, probably due to limitations in the ex vivo MRS technique used [13]. In another study performed with in vivo MRS in a transient MCAO mice model (30 min) at day 1 post-stroke, an increase in lactate and a decrease in NAA were detectable, concomitant with increases in glutamine and decreases in TCr, Myo, alanine, GABA, glutamate, glycine, total choline, glutathione, glucose, phosphatidylethanolamine and taurine [27]. Although both studies show slight differences, maybe due to differences in species or the durability of the ischemia (permanent versus transient), they give an idea of the different metabolic pathways affected during stroke and the complexity of the disease.

On the other hand, in the subacute phase metabolic changes tend to stabilize representing the established infarct. In a permanent MCAO rat model, decreases in the initial levels of lactate, NAA, TCr, glutamate and glutamine were described at day 7 post-stroke [28] and in a transient MCAO rat model (45 min) a gradual increase in mobile lipid signals [ML(–CH_2_)] were described [29].

Our results using multiparametric analysis-derived classifiers in a transient MCAO rat model (90 min) show that only a few metabolites among all possible candidates (already shown to change by work mentioned above) would be suitable as MRS biomarkers for monitoring infarct evolution during the acute and the subacute phase: TCr, Lac + ML(–CH_2_) and ML(–CH_3_).

TCr mostly comprises the resonances of creatine and phosphocreatine. These are involved in the creatine kinase reaction that replenishes consumed ATP. TCr concentration is thought to be highly stable throughout the brain. This is the reason why it is often used as an internal reference to analyze other metabolic changes. However in the infarcted parenchyma, TCr concentration is decreased, probably due to diminished viable cell density [30]. Moreover, our results suggest that TCr levels are not recovered in the subacute phase of infarct.

The Lac and the ML(–CH_2_) resonances at 1.33 ppm appear to increase continuously during the acute and the subacute phase of infarct, but due to the spectral overlapping, they cannot be separated at the echo time used in this study (12 ms) without additional post-processing methods [17]. Lac is a metabolite resulting from the onset of anaerobic metabolism. It is rapidly increased during the first hours post-stroke and diminished thereafter, as brain cells die and perfusion allows extracellular Lac clearance [29]. However, in the subacute phase of infarct, it is ML(–CH_2_) and not Lac that differentially increases, as can be confirmed by the increase in the other functional group of ML, the ML(–CH_3_) at 0.85 ppm. Moreover, this latter resonance is the only significant change detected between the acute and the subacute phase post-stroke. This is in agreement with the in vivo and ex vivo data from our previous work [17] which confirms the good performance of the pattern-recognition software in objectively detecting spectral pattern changes. Thus, ML(–CH_3_) is the most suitable surrogate spectral biomarker detected for the infarct evolution.

ML resonances are not found in the non-infarcted parenchyma, but are associated with many metabolically altered states as proliferative, necrotic/apoptotic or inflammation processes [31]. ML are related to changes in the cell membrane, which are reflected in changes in the composition and dimensions of cytosolic lipid droplets [32, 33] that contain neutral lipids (mainly triacylglycerids and cholesterol esters). Such ML accumulation was described in a rat stroke model [29].

The Brain Regions Classifier was used to distinguish among non-infarcted parenchyma, SVZ and infarcted parenchyma. The SVZ is a thin layer of cells in the wall of the lateral ventricles [34] which cannot be completely isolated from the surrounding tissues using in vivo MRS. However, potential metabolic contributions of these SVZ to the voxel can contribute to its differentiation from other brain regions.

The metabolites that originate the features selected for this Brain Regions classification were TCr and Myo.

TCr is able to differentiate the infarcted parenchyma class from the other two classes. This is consistent with the Infarct Evolution Classifier where TCr was chosen also to differentiate non-infarcted parenchyma from infarcted parenchyma.

Myo is significantly different among all the classes, for this reason it is the only detected surrogate spectral biomarker able to differentiate the SVZ from non-infarcted parenchyma or infarcted parenchyma. Myo is an osmolyte present in the cytoplasm that preserves the osmotic equilibrium during osmotic stress. It is considered a specific marker of glial cells [35]. Myo is altered in cerebral diseases, including stroke [36]. Its decrease in stroke may be related (similarly to TCr) to the loss of viable cells in the infarcted parenchyma [37]. On the other hand, the Myo concentration in the SVZ may be relevant since it is the only metabolite that differentiates between SVZ and non-infarcted parenchyma. Accordingly, a high content of Myo in neural stem cells (NSC) was described [38]. This Myo content of NSC may be related to the fact that NSC are astrocyte-like cells [39]. Furthermore, Myo content in cultured human striatal NSC decreased 48-fold when differentiation was induced [40].

The metabolites proposed as surrogate spectral biomarkers must be validated in larger studies, especially in a clinical setting, to test whether they could be followed-up to detect and monitor changes in the SVZ after a stroke or temporal changes in the infarct evolution.

Moreover, the use of SpectraClassifier as the main approach to identify potential spectroscopic biomarkers could be used in any research question based on MRS data. For example one of its interests could be in the evaluation of the effect of different stroke therapies. Finally, SpectraClassifier is a relatively fast and user-friendly software, which make it adequate for clinical applications [9, 10].

## Conclusions

SpectraClassifier has been used to identify potential spectral biomarkers from infarct evolution (ML) and different brain regions (Myo). This software may be applied in future spectral metabolomic studies in the preclinical or clinical setting.

## Additional files

**Additional file 1.** Training set used for the infarct Evolution classifier. Contains data from 54 processed spectral data vectors corresponding to non-infarcted parenchyma (n=32), acute phase of infarct (n=13) and subacute phase of infarct spectra (n=9).

**Additional file 2.** Test set data used for the Infarct Evolution Classifier. Contains data from 28 processed spectral data vectors corresponding to non-infarcted parenchyma (n=16), acute phase of infarct (n=7) and subacute phase of infarct spectra (n=5).

**Additional file 3.** Training set data used for the Brain Regions Classifier. Contains data from 108 processed spectral data vectors corresponding to non-infarcted parenchyma (n=32), SVZ (n=54) and infarcted parenchyma (n=22).

**Additional file 4.** Test set data used for the Brain Regions Classifier. Contains data from 56 processed spectral data vectors corresponding to non-infarcted parenchyma (n=16), SVZ (n=28) and infarcted parenchyma (n=12).

## Abbreviations

MRS: magnetic resonance spectroscopy
ppm: parts per million
MR: magnetic resonance
SVZ: subventricular zone
MCAO: middle cerebral artery occlusion
UL_2_CA: unit length normalized to the contralateral area
ID: identification number
SFFS: sequential forward feature selection
LDA: linear discriminant analysis
CCC-value: correctly classified cases value
BER-value: balanced error rate value
PPV: positive predictive value
NPV: negative predictive value
AUC: area under curve
ROC: receiver operating characteristic
SD: standard deviation
GABA: γ-aminobutyric acid
NAA: *N*-acetyl aspartate
Lac + ML(–CH_2_): lactate and the methylene of mobile lipids
TCr: total creatine
ML(–CH_3_): methyl of mobile lipids
Myo: myoinositol
NSC: neural stem cells
